# A randomized controlled trial comparing non-selective *versus* selective TIRADS-based cytology in thyroid cancer diagnostics

**DOI:** 10.1093/bjs/znag076

**Published:** 2026-06-20

**Authors:** Jakob Dahlberg, Jeanette Carlqvist, Ann Örtoft, Lilian Hammarstedt, Ekaterina Aula, Mikael Hellström, Erik Elias, Andreas Muth

**Affiliations:** Department of Surgery, Institute of Clinical Sciences, Sahlgrenska Academy, University of Gothenburg, Gothenburg, Sweden; Department of Surgery, Sahlgrenska University Hospital, Gothenburg, Sweden; Department of Radiology, Institute of Clinical Sciences, Sahlgrenska Academy, University of Gothenburg, Gothenburg, Sweden; Department of Radiology, Sahlgrenska University Hospital, Gothenburg, Sweden; Department of Radiology, Northern Älvsborg Hospital, Trollhättan, Sweden; Department of Radiology, Kungälv Hospital, Kungälv, Sweden; Department of Radiology, Southern Älvsborg Hospital, Borås, Sweden; Department of Radiology, Institute of Clinical Sciences, Sahlgrenska Academy, University of Gothenburg, Gothenburg, Sweden; Department of Radiology, Sahlgrenska University Hospital, Gothenburg, Sweden; Department of Surgery, Institute of Clinical Sciences, Sahlgrenska Academy, University of Gothenburg, Gothenburg, Sweden; Department of Surgery, Sahlgrenska University Hospital, Gothenburg, Sweden; Department of Surgery, Institute of Clinical Sciences, Sahlgrenska Academy, University of Gothenburg, Gothenburg, Sweden; Department of Surgery, Sahlgrenska University Hospital, Gothenburg, Sweden

## Abstract

**Introduction:**

Several ultrasound risk stratification systems have been developed mainly with the aim of identifying benign lesions and thereby avoiding unnecessary fine needle aspiration (FNA) cytology. This randomized controlled trial assessed whether the use of an ultrasound risk stratification system improved identification of lesions requiring surgical treatment.

**Material and methods:**

This was a multi-centre, unblinded and interventional randomized trial comparing selective and non-selective FNA in Western Sweden. Patients were randomized to either selective cytology according to EU-TIRADS criteria or non-selective cytology.

**Results:**

A total of 195 patients were included, 93 in the non-selective group and 102 in the selective group, between February 2022 and December 2023. The frequency of nodules with Bethesda category IV–VI (primary outcome) was higher in the selective group (26% *versus* 13%, *P* = 0.039). The rate of malignancy (secondary outcome) was similar in both groups; 8% in the selective group *versus* 5% in the non-selective group. The frequency of patients undergoing cytology was reduced from 83% in the non-selective group to 71% in the selective group. Considering only patients with at least one nodule yielding EU-TIRADS 3 or higher, cytology was omitted in 7% of patients in the selective group, whereas no cytology was omitted in the non-selective group.

**Conclusion:**

This randomized controlled trial supports the use of EU-TIRADS to correctly select neoplastic nodules for FNA without missing thyroid cancer. The proportion of patients where FNA can be safely omitted using EU-TIRADS may however be exaggerated, indicating a need for further refinement of risk stratification systems for thyroid cancer diagnostics.

The trial was registered at ClinicalTrials.gov (NCT05583097).

## Teaser

In a thyroid cancer diagnostic programme, ultrasound-based risk stratification with EU-TIRADS correctly omits cytology of benign disease without compromising cancer diagnostics. However, in a population with mainly palpable goitre, the effect size is modest.

## Introduction

Fine needle aspiration cytology (FNA) has been standard of care for evaluation of thyroid nodule malignancy risk for decades^1^. Ultrasound is the first-line radiological modality because it is non-invasive, readily available, cost effective, and the detection rate with high-resolution ultrasound is close to what has been observed in autopsy studies. During the last 25 years, the use of cervical ultrasound in the clinical work-up of thyroid disease has gradually increased, leading to a higher rate of thyroid nodule detection. Although thyroid nodules are common in the adult population^2,3^, the vast majority are benign^4^. To assess risk of thyroid malignancy, several ultrasound risk stratification systems (RSS) have been developed^5–7^ since the original version of TIRADS (Thyroid Imaging Report and Data System) was published in 2009 by Horvath *et al*.^8^. The original version of TIRADS was difficult to implement in everyday clinical practice due to its complexity. More recent versions of TIRADS, including EU-TIRADS^5^, have been developed to facilitate implementation in clinical practice, with the aim to reduce unnecessary FNAs.

Both retrospective and prospective studies have demonstrated the performance of ultrasound RSS^9–11^ showing that it is possible to identify benign lesions and safely omit FNA. No system has been shown to be superior, although several studies comparing different ultrasound RSS have been published^12–14^. No RCTs comparing thyroid RSS to non-selective FNA have been published.

In 2017, a national standardized care pathway (SCP) for thyroid cancer was introduced in Sweden. The rationale was to ensure uniform and swift work-up including ultrasound and FNA for patients with signs or symptoms that could indicate thyroid cancer. In Western Sweden, it was decided to introduce a thyroid ultrasound RSS with selective FNA, performed as a ‘one-stop-shop’ procedure. Subsequently, EU-TIRADS was implemented in the Swedish National Guidelines for thyroid cancer in 2021. This multi-centre regional study aimed to investigate whether EU-TIRADS identifies benign nodules and thus correctly omits FNA. Primary outcome was the proportion of cytology suspicious of thyroid cancer (Bethesda IV–VI). In the selective group, low-risk nodules did not undergo cytology and therefore the frequency of cytologically suspicious nodules was expected to be higher in this group. Secondary outcome was the rate of malignancy, and it was hypothesized that there was no difference between the two groups.

## Material and methods

### Clinical setting

This was a regional, multi-centre (four units), unblinded, interventional prospective randomized trial comparing selective and non-selective FNA of thyroid nodules in the thyroid cancer diagnostic programme in Western Sweden. Western Sweden is one of six healthcare regions in Sweden with a population of approximately 1.7 million people (year 2023). During the study period, diagnostic work-up according to the SCP was available only by the public healthcare system in Western Sweden. Participating units consisted of one university hospital (Sahlgrenska University Hospital (SU)), two regional hospitals (Northern Älvsborg Hospital (NAL) and Southern Älvsborg Hospital (SÄS)), and one local hospital (Kungälv Hospital (KS)), covering a geographical area with approximately 1.5 million inhabitants.

### Objective

The primary aim of this study was to evaluate the ability of the EU-TIRADS RSS to select suspicious nodules for FNA. Primary outcome was the frequency of FNA suspicious of or showing thyroid cancer (Bethesda IV–VI), as this would elicit further diagnostics or treatment. It was hypothesized that the proportion of Bethesda IV–VI would be higher in the intervention (selective) than in the control (non-selective) group. A secondary aim was to assess the risk of missing thyroid cancer using EU-TIRADS. The rate of malignancy was defined as number of patients with postoperative thyroid cancer diagnosis divided by total number of patients undergoing examination in each group. It was hypothesized that the rate of malignancy did not significantly differ between groups.

### Intervention

Patients were randomized 1:1 to an intervention (selective) or a control (non-selective) group. All low-risk and intermediate-risk (EU-TIRADS ≤4) nodules ≥1 cm in both groups were recorded in the case report form (CRF), as were nodules larger than 0.5 cm in diameter when classified EU-TIRADS 5. Indication for FNA in the intervention group was identical to EU-TIRADS recommendations^5^. In the control group, all nodules >1 cm scoring EU-TIRADS 4 or lower underwent FNA in up to five nodules per patient. Nodules scoring EU-TIRADS 5 underwent FNA regardless of size if technically possible. If the patient had more than five nodules, the five most suspicious underwent FNA.

### Inclusion and exclusion criteria

All patients in Western Sweden, 18 years of age or older, referred to the radiology department for ultrasound-guided FNA due to signs or symptoms that could indicate thyroid cancer according to the SCP were eligible for enrolment in the study. Signs and symptoms that qualified a patient for referral to the radiology department according to the SCP were: (1) previously undetected palpable nodule in the thyroid, (2) growing palpable nodule in the thyroid, and/or (3) an incidental focal PET-positive uptake in the thyroid. Exclusion criteria were previous history of thyroid cancer or previous thyroid surgery.

### Power calculation and patient inclusion

A power calculation based on institutional data was performed to calculate the number of patients needed. A historic control group in which patients underwent non-selective FNA without ultrasound risk stratification between 2013 and 2017 was used. Initially Bethesda III was included in cytology suspicious for malignancy based on our experience and clinical management. Thus, Bethesda III was included in the primary endpoint when trial was first registered at ClinicalTrial.gov. However, before commencement of the trial the second edition of the Bethesda classification was implemented along with other alterations to cytology evaluation in clinical routine. Consequently, Bethesda III became much less influential in clinical management and was removed from the primary endpoint. In historical data, the frequency of Bethesda IV–VI was 46% in operated patients. Preliminary data (not published) from patients referred according to SCP criteria and examined with selective FNA according to EU-TIRADS between March 2018 and October 2019 showed frequency of Bethesda IV–VI of 69%, yielding an effect size of 23%. Power was estimated at 0.8 and significance level 0.05, requiring a total of 149 observations. In order not to be underpowered and compensate for dropouts, it was decided to include 200 patients in total.

Secondary outcome was rate of malignancy, and it was hypothesized that this would not be significantly different in the two groups. A non-inferiority power calculation showed that a minimum of 600 patients would be required, which was not considered possible to achieve in reasonable time.

Inclusion of patients was planned between February 2022 and September 2023. The study period was prolonged by 3 months, as only 170 patients had been included by September 2023. In December 2023, 195 patients were randomized (five less than planned but more than estimated by the power calculation) and it was decided to close inclusion due to slow recruitment.

### Randomization

Oral and written informed consent were obtained before examination. Patients were randomized 1:1, in random permutated blocks using an online tool (www.sealedenvelope.com/simple-randomiser/v1/lists) by staff at the radiology department prior to examination.

### Ultrasound examination

Patients were referred from primary care and hospital-based services. Primary examination of included patients was between February 2022 and December 2023. Patients were examined at the radiology department at four participating public hospitals.

Ultrasound was performed at the four centres using a Canon Aplio i800 ultrasound scanner (Canon Medical Systems Corp., Otawara, Japan) equipped with a 14L5 linear array transducer probe (Toshiba Medical Systems, Tochigi, Japan) (SU), Canon Aplio i700 ultrasound scanner (Canon Medical Systems Corp., Otawara, Japan) equipped with a 14L5 linear array transducer probe (Toshiba Medical Systems, Tochigi, Japan) (NÄL), and Philips EPIQ 7, Philips Ultrasound, Bothell, WA 98021, USA (KS and SÄS). Thyroid nodules were classified according to the EU-TIRADS system^5^. Ultrasound images of relevant nodules with measurements in three dimensions as well as size of the thyroid gland were stored together with 10 MHz transverse scan sequences over both thyroid lobes to enable secondary assessment if needed. In addition to routine documentation of thyroid nodules and cervical lymph nodes, a CRF was filled in for study purposes by the radiologist directly after examination.

### Fine needle aspiration cytology

All US examinations were carried out with ‘one-stop’ FNA. In the control group, according to study protocol, all solid or partially solid nodules (EU-TIRADS ≥3) that were >1 cm underwent FNA. Nodules <1 cm scoring EU-TIRADS 5 underwent FNA if possible. In the intervention group, nodules underwent FNA according to EU-TIRADS criteria^5^.

Nodule puncture was performed with a 22G needle with vacuum applied using a syringe. All FNAs were ultrasound-guided and performed by the radiologist and evaluated by two board-certified cytologists. The 2017 Bethesda system for reporting thyroid cytology was used consistently throughout the study^15^.

### Clinical workup

Clinical management of patients undergoing FNA was determined by an endocrine surgeon at SU or NÄL based on results from ultrasound and FNA. All surgical procedures and histopathologic examinations were performed at SU and NÄL.

Indication for surgery included FNA suspicious for or showing thyroid cancer, benign goitre and hyperthyroidism. In short, patients with Bethesda category IV or higher were offered surgery within 31 days, either hemi- or total thyroidectomy according to Swedish national guidelines. Central lymph node dissection was performed in patients with clinically suspicious lymph nodes or FNA verified lymph node metastasis or primary tumour >4 cm. Lateral lymph node dissection was performed when US suggested metastatic spread, usually confirmed by FNA. Nodules with indeterminate FNA (Bethesda I and III) and nodules yielding EU-TIRADS 5/Bethesda II were referred to follow-up by US and FNA after 1–6 months unless surgery was indicated due to compression symptoms.

Microcarcinomas, detected postoperatively and not recorded in the CRF, were not included in the statistical analysis. Borderline tumours were regarded as benign.

All patients with a postoperative cancer diagnosis underwent secondary review of FNA and histopathology at the department of pathology at SU and received further treatment recommendations based on discussion at a multidisciplinary tumour board.

### Statistics

Descriptive statistics are presented as total numbers (%). The chi-square test, Wilcoxon rank sum test and Students *t*-test were used for comparisons between groups. R, version 4.3.2 (https://www.R-project.org/), was used for calculations.

### Ethical considerations

The study was approved by the Swedish Ethical Review Authority (2021-01490).

## Results

### Study cohort

**Figure 1:**
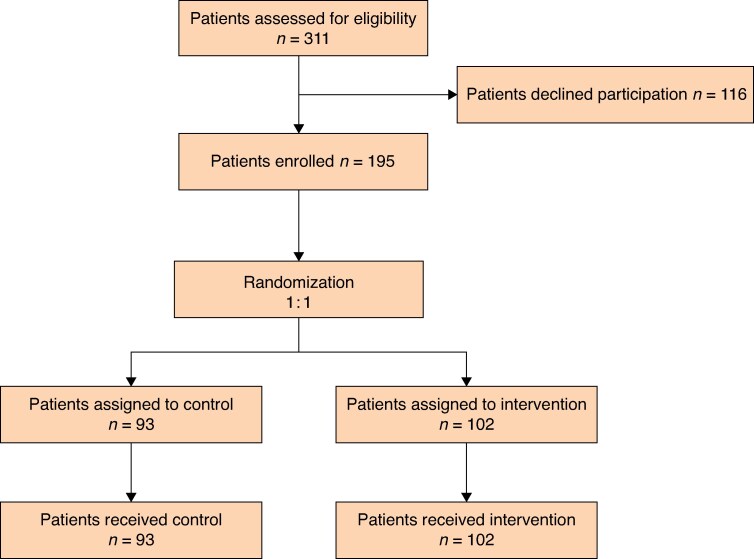
Consort diagram of included and excluded patients

Altogether 311 patients were offered inclusion in the study. One hundred and sixteen patients declined participation and of the 195 patients who accepted to participate, 93 patients (48%) with 150 nodules (58.1%) were randomized to the control (non-selective) group and 102 (52%) patients with 108 (41.8%) nodules to the intervention (selective) group. There were no dropouts among included patients. *Figure 1* shows a consort diagram of included and excluded patients. Basic characteristics of patients, participating radiology units and indication for referral and referring unit are shown in *Table 1*.

**Table 1:** Patient characteristics, performing units, and indications for examination

	Non-selective	Selective	Total
**Patients, *n* (%)**	93 (47.7)	102 (52.3)	195
Female, *n* (%)	69 (74.2)	81(79.4)	150 (76.9)
Male, *n* (%)	24 (25.8)	21 (20.5)	45 (23.1)
Mean age, years (s.d.)	58 (15)	55 (15)	56 (15)
**Units performing examinations, *n* (%)**			
SU*	40 (43.0)	45 (44.1)	85 (43.6)
NÄL*	29 (31.3)	30 (29.4)	59 (30.3)
Kungälv Hospital	24 (25.8)	25 (24.5)	49 (25.1)
SÄS*	0 (0)	2 (2.0)	2 (1.0)
**Indication, *n* (%)**			
Palpable nodule/goitre symptoms	60 (64.5)	74 (72.5)	134 (68.7)
PET positive uptake	4 (4.3)	4 (3.9)	8 (4.1)
Incidental imaging (CT/MR/ultrasound)	29 (31.2)	24 (23.5)	53 (27.2)
**Referral from, *n* (%)**			
Primary care	51 (54.8)	58 (56.9)	109 (55.9)
Hospital-based	42 (45.2)	44 (43.1)	86 (44.1)

^*^SU, Sahlgrenska University Hospital; NÄL, Northern Älvsborg Hospital; SÄS, Southern Älvsborg Hospital.

### Use of EU-TIRADS led to fewer fine needle aspiration cytology examinations per patient

In total 258 nodules were found at ultrasound. The mean nodule size and the distribution between the two randomized groups are shown in *Table 2*. The frequency of patients undergoing FNA was 83% (*n* = 77) in the control (non-selective) group compared to 71% (*n* = 72) in the intervention (selective) group. Considering only patients with solid or partially solid nodules (EU-TIRADS ≥3), FNA was omitted according to study protocol in 9% of the patients (*n* = 7) in the intervention group and in no patient in the control group. In the intervention group, a mean of 0.9 FNA/patient was performed compared to 1.5 in the control group (*P* = 0.002, *Table 2*).

**Table 2:** Evaluation of patients and nodules

	Non-selective	Selective	Total	*P*
Nodules, *n* (%)	150 (58.1)	108 (41.9)	258 (100)	
Mean size, mm (s.d.)	24 (14)	29 (13)	26 (14)	
Patients undergoing FNA, *n* (%)	77 (82.8)	72 (70.6)	149 (76.4)	
Highest Bethesda category per patient, *n* (%*)				
No FNA	16	30	46	
Bethesda I	17 (22.1)	16 (22.2)	33 (22.1)	
Bethesda II	43 (55.8)	32 (44.4)	75 (50.3)	
Bethesda III	7 (9.1)	5 (6.9)	12 (8.1)	
Bethesda IV	7 (8.9)	15 (20.8)	22 (14.8)	
Bethesda V	1 (1.3)	3 (4.2)	4 (2.7)	
Bethesda VI	2 (2.6)	1 (1.4)	3 (2.0)	
Number of FNAs/patient	143/93 = 1.5	96/102 = 0.9	1.2	0.002
Cancer prevalence (number of patients), *n* (%)	5 (5.4)	7 (6.9)	12 (6.2)	

^*^Only patients undergoing FNA included in calculation. FNA, fine needle aspiration.

### Use of EU-TIRADS led to less fine needle aspiration cytology examinations for benign lesions

**Figure 2:**
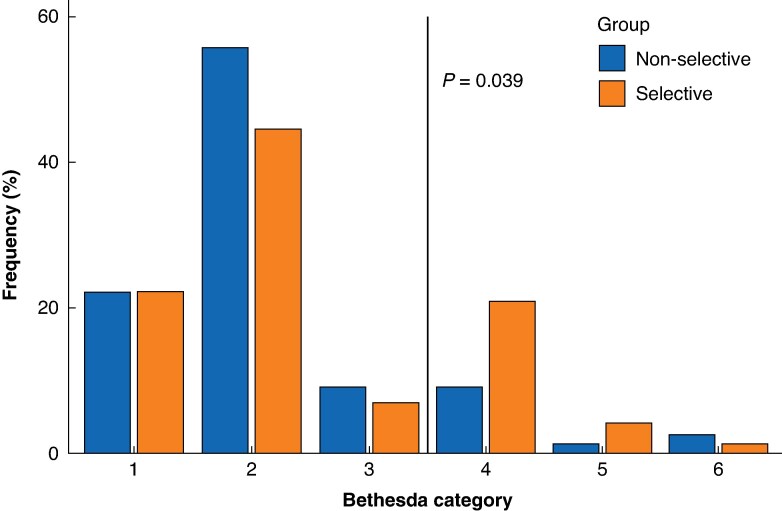
**Distribution of maximum Bethesda category per patient based on randomization between non-selective (control) and selective (intervention) cytology**. Horizontal line divides patients with cytologically benign or low-risk nodules to the left (Bethesda I–III) and patients with neoplastic nodules to the right (Bethesda IV–VI). Patients undergoing selective cytology had a significantly higher frequency of neoplastic nodules (*P* = 0.039)

EU-TIRADS RSS for FNA, selected patients with neoplastic lesions (Bethesda IV–VI) to a higher degree compared to the non-selective group (26% *versus* 13%, *P* = 0.039), which was the primary endpoint of the trial (*Table 2* and *Fig. 2*). The number of nodules in the intervention group yielding Bethesda IV–VI at primary examination was 21 (20%) compared to 10 (7%) for the control group (*P* = 0.002, *Table S1*).

### Surgical outcomes

Of the 195 included patients, 46 (23.6%) underwent surgery. Indication for surgery was at ultrasonography and/or FNA suspicious of or showing malignancy in 20 of 30 (67%) patients in the intervention group and 10 of 16 (63%) patients in the control group. No patients were operated due to toxic goitre. In terms of the secondary endpoint of the trial, histopathology showed thyroid cancer in 8 (8%) *versus* 5 (5%) patients in the intervention and control groups respectively. In the intervention group, no patients were diagnosed with a microcarcinoma, whereas one patient in the control group had papillary thyroid cancer microcarcinoma (EU-TIRADS 4, Bethesda VI). Four patients (three in the intervention and one in the control group) had incidental papillary thyroid cancer microcarcinomas upon final histopathology, not recorded in the CRF and thus not included in the statistical calculations.

### Repeat examinations

In total, 42 patients underwent repeat examination, 19 (19%) in the intervention and 23 (25%) in the control groups. Of these, eight patients finally underwent surgery due to suspicion of malignancy at repeat FNA. Histopathology showed benign results except in one case of incidental papillary microcarcinoma that was not the indication for surgery.

The mean time between first and second examination was 15 months.

## Discussion

In this randomized trial of EU-TIRADS-based selective (intervention) *versus* non-selective (control) ultrasound-guided FNA in a thyroid cancer diagnostic system, there were three main findings. The proportion of patients with FNA suspicious for or showing thyroid cancer was significantly higher in the intervention group compared with the control group. This difference was even more pronounced when accounting for individual nodules. Furthermore, the study demonstrated a 39% reduction of FNA, indicating a higher selectivity when using EU-TIRADS. Second, the proportion of patients with solid lesions where FNA was omitted according to EU-TIRADS was 9% in the intervention group. Finally, the rate of malignancy was similar in the intervention and control groups, supporting the secondary hypothesis of retained diagnostic sensitivity using of EU-TIRADS.

The primary aim of this study was to evaluate the performance of EU-TIRADS in selecting neoplastic nodules for FNA investigation. The frequency of Bethesda category IV–VI lesions was significantly higher in the intervention (9%) compared to the control (5%) group, when comparing individual nodules. This is the first RCT to show that EU-TIRADS improves the selection of nodules for FNA.

When analysing the effect of EU-TIRADS on a patient level, the frequency of patients with cytologically suspicious or malignant findings in the intervention group was higher although less pronounced compared to analysis of individual nodules. It could be speculated that the power calculation was based on a cohort that had a higher degree of symptomatic palpable nodules. In the current cohort, even if most patients had palpable goitre with a mean nodule size of 26 mm, patients with less obvious symptoms were also referred for suspected thyroid cancer and included. Consequently, a higher degree of thyroid ultrasound exams showed no focal lesions or ultrasound signs of thyroiditis resulting in EU-TIRADS 1. For these patients an ultrasound RSS does not contribute to patient management. This also raises the question about what patients should be referred to cervical ultrasonography, and which groups benefit most from ultrasound RSS. The Swedish national guidelines for referring patients to cervical ultrasonography according to ‘standardized care pathway’^16^ is an attempt to reduce the number of unnecessary examinations and possibly unnecessary treatments.

The frequency of FNA performed per patient was significantly lower in the group of patients randomized to selective FNA according to EU-TIRADS. This finding is not surprising because all or most ultrasound RSS including EU-TIRADS have been developed mainly to identify benign nodules so that FNA may be safely avoided. The many different ultrasound RSS currently used have a varying degree of ‘ruling out’ benign nodules^13^, although the decision to recommend FNA is based on similar features, for example echogenicity, shape, margins, presence of calcifications and nodule size. In the present study, EU-TIRADS identified 9% of the patients with solid or mixed solid nodules >1 cm in which FNA was not indicated or performed. The development of ultrasound RSS should strive to improve cost effectiveness and reduce invasive procedures such as FNA, while retaining diagnostic sensitivity. It has been reported that up to 50% of nodules >1 cm detected on ultrasound may not be subject to FNA^13^, which is a substantially higher figure compared to the data in the current study. This may be explained by differences in patient cohorts in relation to RSS criteria for avoiding FNA. Most ultrasound RSS and thyroid nodule management guidelines generally recommend no FNA for lesions <1 cm, and when applying EU-TIRADS, only nodules between 1 and 2 cm are within the range of choosing between FNA or no FNA. In the current material, 60% of the nodules were larger than 2 cm and thus the decision to perform FNA was not affected by EU-TIRADS classification. Other ultrasound RSS such as ACR TIRADS have higher cut-off levels for performing FNA (for example 2.5 cm for TR3/Mildly suspicious)^6^.

The rate of malignancy was similar in both groups, indicating that thyroid cancer was not missed using EU-TIRADS. However, this must be interpreted with caution as the difference of FNA use was only 9% between the groups for solid lesions. When omitting FNA there is a risk of missing a thyroid cancer that eventually will cause symptoms. This is especially true for follicular thyroid cancer (FTC), as it is difficult to distinguish FTC from a follicular thyroid adenoma. The ultrasound features included in most ultrasound RSS are relevant mainly for papillary thyroid cancer and ultrasound features associated with FTC such as characteristics of the halo, macro-calcifications and rim calcifications^17^ do not affect EU-TIRADS classification. In a country like Sweden where patients with low- or intermediate-risk thyroid nodules rarely are engaged in structured ultrasound follow-up programmes, it is probably not wise to recommend a higher cut-off level for FNA. Thus, FNA remains an important tool in thyroid nodule evaluation.

Modifications from the first TIRADS system^8^ have focused on simplifying the stratification system to enable clinical implementation. This comes at the expense of a lower specificity and the present trial illustrates that the performance of EU-TIRADS is affected by the patients examined. The experience of the examiner also seems to affect outcome^18^. Moving on, AI-guided pattern recognition could potentially improve diagnostic performance. A recently published review concluded that AI or deep learning may improve thyroid nodule evaluation^19^.

This RCT supports the use of EU-TIRADS to correctly select neoplastic nodules for FNA without missing thyroid cancer. However, only 10% of patients were freed from suspicion of malignancy based on EU-TIRADS alone without need for FNA, thus indicating a need for further refinement of risk stratification systems for thyroid cancer diagnostics.

## Supplementary Material

znag076_Supplementary_Data

## Data Availability

Data may be available at the premises upon reasonable request.
