# Supplementary material for: A randomized controlled trial comparing non-selective versus selective TIRADS-based cytology in thyroid cancer diagnostics
Source: Br J Surg. 2026 Jun 20;113(7):znag076. doi: 10.1093/bjs/znag076 (PMC13367576; doi:10.1093/bjs/znag076)
Supplement: znag076_Supplementary_Data [file znag076_supplementary_data.zip › Supplementary_material.docx]

# A randomized controlled trial comparing non-selective versus selective TIRADS-based cytology in thyroid cancer diagnostics

Jakob Dahlberg^1^, Jeanette Carlqvist^2^, Ann Örtoft^3^, Lilian Hammarstedt^4^, Ekaterina Aula^5^, Mikael Hellström^2^, Erik Elias^1^, Andreas Muth^1^

1. Department of Surgery, Institute of Clinical Sciences, Sahlgrenska Academy, University of Gothenburg, Gothenburg, Sweden and Department of Surgery, Sahlgrenska University Hospital, Gothenburg, Region Västra Götaland, Sweden
2. Department of Radiology, Institute of Clinical Sciences, Sahlgrenska Academy, University of Gothenburg, Gothenburg Sweden and Department of Radiology, Sahlgrenska University Hospital, Region Västra Götaland, Gothenburg, Sweden
3. Department of Radiology, Northern Älvsborg Hospital, Trollhättan, Region Västra Götaland, Sweden
4. Department of Radiology, Kungälv Hospital, Kungälv, Region Västra Götaland, Sweden
5. Department of Radiology, Southern Älvsborg Hospital, Borås, Region Västra Götaland, Sweden

Correspondence: Dr Jakob Dahlberg, Department of Surgery, Sahlgrenska University Hospital, Blå Stråket 5, 413 45 Gothenburg, Sweden. [jakob.dahlberg@gu.se](mailto:jakob.dahlberg@gu.se)

ORCID 0000-0001-9097-8691

**Supplementary Materials - Index**

| **Supplementary Figures and Tables** |  |
| --- | --- |
| Supplementary table 1 | *Page 1* |
