# Supplementary material for: A randomized controlled trial comparing non-selective versus selective TIRADS-based cytology in thyroid cancer diagnostics
Source: Br J Surg. 2026 Jun 20;113(7):znag076. doi: 10.1093/bjs/znag076 (PMC13367576; doi:10.1093/bjs/znag076)
Supplement: znag076_Supplementary_Data [file znag076_supplementary_data.zip › Supplementary_table_1.docx]

Supplementary table1. FNA – fine needle aspiration cytology.

| **Distribution of Bethesda category per nodule, n (%)** | Non selective | Selective | Total |
| --- | --- | --- | --- |
| No FNA | 8 (5) | 12 (11) | 20 (8) |
| Bethesda I | 36 (24) | 20 (19) | 56 (22) |
| Bethesda II | 82 (55) | 48 (44) | 130 (50) |
| Bethesda III | 14 (9) | 7 (6) | 21 (8) |
| Bethesda IV | 6 (4) | 15 (14) | 21 (8) |
| Bethesda V | 1 (1) | 5 (5) | 6 (2) |
| Bethesda VI | 3 (2) | 1 (1) | 4 (2) |
